# Use of plasma metabolomics to analyze phenotype-genotype relationships in young hypercholesterolemic females[S]

**DOI:** 10.1194/jlr.M088930

**Published:** 2018-09-28

**Authors:** Xiang Zhang, Antoine Rimbert, Willem Balder, Aeilko Having Zwinderman, Jan Albert Kuivenhoven, Geesje Margaretha Dallinga-Thie, Albert Kornelis Groen

**Affiliations:** Departments of Experimental Vascular Medicine* University of Amsterdam, Amsterdam, The Netherlands; Clinical Epidemiology, Biostatistics, and Bioinformatics,** Amsterdam University Medical Center, University of Amsterdam, Amsterdam, The Netherlands; Department of Pediatrics, Section Molecular Genetics,† University of Groningen, Groningen, The Netherlands; Department of Pediatrics,†† University Medical Center Groningen, University of Groningen, Groningen, The Netherlands; Department of Cardiology,§ Jeroen Bosch Hospital, ’s-Hertogenbosch, The Netherlands

**Keywords:** hypercholesterolemia, triglyceride, low density lipoprotein, genetics, metabolomics

## Abstract

Hypercholesterolemia is characterized by high plasma LDL cholesterol and often caused by genetic mutations in LDL receptor (*LDLR*), *APOB*, or proprotein convertase subtilisin/kexin type 9 (*PCSK9*). However, a substantial proportion of hypercholesterolemic subjects do not have any mutations in these canonical genes, leaving the underlying pathobiology to be determined. In this study, we investigated to determine whether combining plasma metabolomics with genetic information increases insight in the biology of hypercholesterolemia. For this proof of concept study, we combined plasma metabolites from 119 hypercholesterolemic females with genetic information on the LDL canonical genes. Using hierarchical clustering, we identified four subtypes of hypercholesterolemia, which could be distinguished along two axes represented by triglyceride and large LDL particle concentration. Subjects with mutations in *LDLR* or *APOB* preferentially clustered together, suggesting that patients with defects in the LDLR pathway show a distinctive metabolomics profile. In conclusion, we show the potential of using metabolomics to segregate hypercholesterolemic subjects into different clusters, which may help in targeting genetic analysis.

Hypercholesterolemia due to a high concentration of plasma LDL cholesterol has been shown to be a causal factor in accelerating atherosclerosis in a plethora of studies (1, 2). The liver plays a pivotal role in the regulation of cholesterol metabolism. It secretes cholesterol packaged in VLDL particles that are subsequently converted into IDL and LDL particles, largely by the action of different lipases in the periphery (3). A key step in the uptake of cholesterol is the internalization of LDL via the LDL receptor (LDLR) (4). Mutations in the LDLR as well as mutations in genes encoding APOB or proprotein convertase subtilisin/kexin type 9 (PCSK9) are causally related with hypercholesterolemia (5). These genetic mutations, however, do not explain all hypercholesterolemic cases. For instance, in the UK pilot cascade project, 403 of 635 (63.5%) hypercholesterolemic subjects did not have mutations in *LDLR*, *APOB*, or *PCSK9* (6). In a recent large scale study designed to evaluate the prevalence of a familial hypercholesterolemia (FH) mutation among individuals with severe hypercholesterolemia (7), only 24 of 1,386 subjects with LDL cholesterol above 5 mmol/l were identified to have mutations in these three canonical genes. Although the prevalence of genetically defined hypercholesterolemia varies across studies (8), a substantial proportion of hypercholesterolemic subjects do not have mutations in *LDLR*, *APOB*, or *PCSK9*. A major reason for this finding could be the presence of disease-causing mutations in other genes involved in cholesterol homeostasis either affecting the LDLR pathway or other yet to be defined mechanisms. Interestingly, whole exome sequencing of a cohort with FH subjects without mutations in *LDLR*, *APOB*, and *PCSK9* did not identify novel causal mutations (9).

Recently, we analyzed a cohort of 119 young females with plasma LDL cholesterol above the 99th percentile for their age. In 20 hypercholesterolemic females, we identified 12 causal heterozygous mutations in *LDLR* and one causal heterozygous mutation in *APOB* (10). In the 99 remaining females, we found eight subjects carrying a variant in *LDLR* or *APOB* with unknown clinical significance (10). This left us with 91 females that suffered from hypercholesterolemia caused by either a polygenic (11) or epigenetic (12) mechanism, or the presence of a pathogenic variant in yet unknown genes. To get further insight into the underlying pathobiology of hypercholesterolemia of unknown origin, we performed plasma metabolite analysis on all of the 119 hypercholesterolemic females. We hypothesized that mutations in genes belonging to the same metabolic pathway (e.g., the LDLR pathway) should render a similar plasma metabolome. This analysis differentiated four subgroups, which could be distinguished along two axes represented by plasma triglyceride and large LDL particle concentration.

## MATERIALS AND METHODS

### Participants

The selection of the participants (N = 119) in this study is described in detail elsewhere (10). In brief, these women were apparently healthy, aged 25–40 years, and had a plasma LDL cholesterol level above 4.7 mmol/l. Exclusion criteria were diagnosis of cardiovascular disease (e.g., myocardial infarction, stroke, or coronary surgery), diabetes mellitus, use of lipid-lowering drugs, or having aberrant thyroid, liver, or kidney function. The study protocol was approved by the Medical Ethical Committee of the University Medical Center Groningen in The Netherlands and all participants provided written informed consent.

### Next generation sequencing

With a custom target sequencing array developed based on the SureSelect capture system, we sequenced the coding regions of 11 genes, including *LDLR*, *APOB*, *PCSK9*, *LDLRAP1*, *APOE*, *ABCG5*, *LIPA*, *STAP1*, *MTTP*, *ANGPTL3*, and *SAR1B* to assess a monogenic cause of hypercholesterolemia. If a mutation had a minor allele frequency below 0.1% in the Genome of the Netherlands (13), it was considered a rare mutation. Mutations that are verified to cause hypercholesterolemia were listed in our previous publication (10).

Detection of copy number variations was performed using Copy Number Variation Detection in Next-generation Sequencing Gene (CoNVaDINGs) panels (14). Detected copy number variations were validated using either multiplex ligation-dependent probe amplification or by long-range PCR or real-time PCR (10).

### Genetic risk score calculation

To study a possible polygenic cause of hypercholesterolemia, we calculated the weighted genetic risk score (wGRS). The Global Lipid Genetic Consortium meta-analysis of genome-wide association studies identified 95 loci affecting LDL cholesterol concentration (15). Among these loci, 12 SNPs had the highest power to discriminate between FH mutation-negative individuals and the general population (11, 16). For each individual, we calculated the wGRS using the weighted sum of the risk allele (the LDL cholesterol-raising allele) (10). The weights used were the corresponding per-allele effect in plasma LDL cholesterol changes reported by the Global Lipid Genetic Consortium (15).

### Lifestyle score calculation

To investigate the association between lifestyle and plasma metabolome in hypercholesterolemic females, we used a recently described healthy lifestyle score (17). Points were given for the major lifestyle parameters, including smoking status and eating habits. The details were described in our previous publication (10). In short, a maximum of four points reflects a very healthy lifestyle: the smaller the score, the less healthy the lifestyle. The minimum point is zero.

### Metabolite measurements

Fasting plasma samples were routinely collected by Lifelines (www.lifelines.nl) and stored at −80°C until analysis on the Nightingale metabolomics platform (Nightingale Health, Finland). This platform includes 225 metabolic features, including lipids, lipoproteins, fatty acids, amino acids, and glycolysis precursor molecules (listed on https://nightingalehealth.com/biomarkers), using a NMR spectroscopy platform (18, 19).

### Statistical analysis

To explore the subtypes of hypercholesterolemia, we performed hierarchical clustering based on the plasma metabolomics data. Because the metabolomics data contains measurements of different units, we first scaled the data so that every variable had mean 0 and standard deviation 1. Next, we ran the hierarchical clustering with the function, hclust, from R (https://cran.r-project.org/). We used Euclidean distance as the dissimilarity measure and complete linkage as the similarity measure between the clusters. The dendrogram was made by using the ggdendro and ggplot2 (20) R package. Finally, we cut the dendrogram into four clusters by using the cutree function in R.

To identify the cluster corresponding to hypercholesterolemia due to defects in the LDLR pathway, we performed principal component analysis (PCA) on the metabolomics data. Because the data contains measurements of different units, we converted the metabolomics data into ranks, so that every metabolite had a value ranging between 1 and 119. We then calculated the covariance matrix and performed eigenvector decomposition. Entries of every eigenvector are also called loadings. Based on the loadings, we identified metabolites that most correlated to the first and second principal components (PCs) by calculating the Spearman correlation coefficients.

To evaluate associations between genetic risk/lifestyle scores and metabolite concentrations, we applied a nonparametric method, namely, the Kendall’s tau correlation test. We reported the Kendall’s tau correlation coefficient and *P* value. A *P* value below 0.05 is considered significant.

## RESULTS

**Fig. 1.**
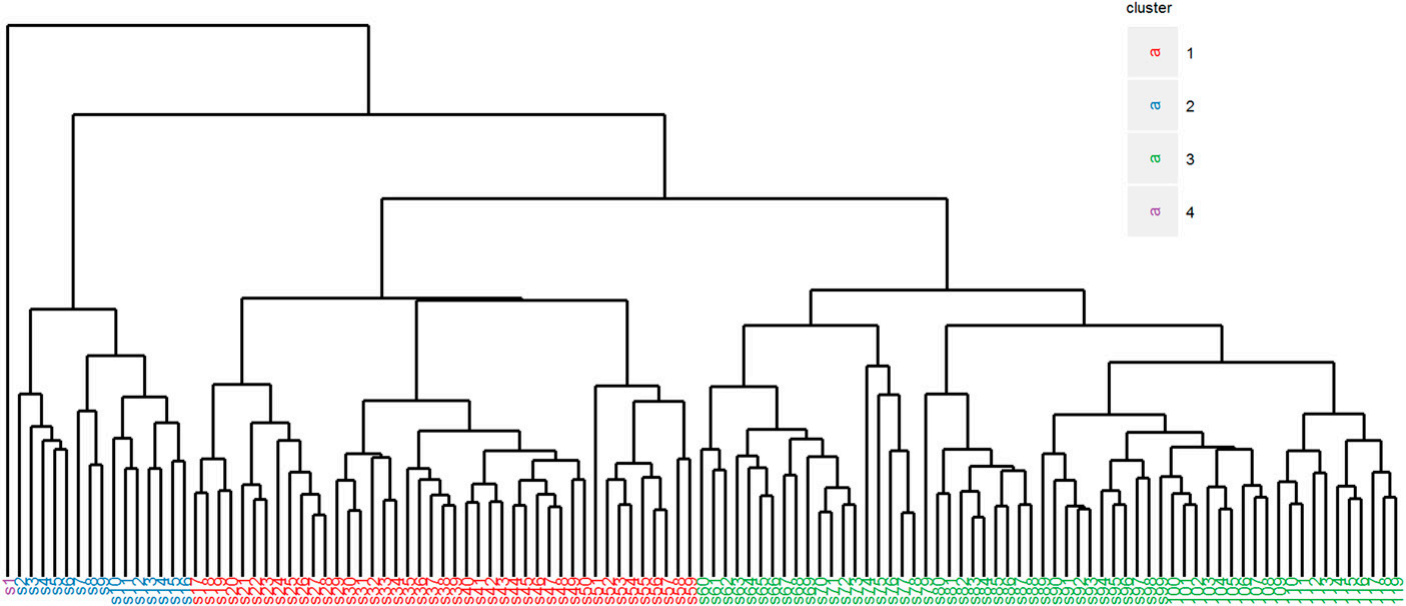
Hierarchical clustering of plasma metabolomics data derived from 119 hypercholesterolemic females. Euclidean distance was used as the dissimilarity measure and complete linkage was used as the dissimilarity measure between the clusters.

A group of 119 young women with hypercholesterolemia, defined as plasma LDL cholesterol levels above the 99th percentile for their age, was selected from the Lifelines cohort. The baseline characteristics are presented in **Table 1**. To analyze the underlying pathobiology of the hypercholesterolemic phenotype, plasma metabolomics was performed using the Nightingale platform. Although the absolute values measured in the Nightingale platform are lower than the conventional measured plasma lipids, the two measurements showed a similar pattern (Table 1). A summary of all the results of metabolite analysis is presented in supplemental Table S1. Hierarchical clustering analysis of the metabolomics data set revealed three main clusters and one cluster containing only one sample (**Fig. 1**). The size of clusters 1, 2, 3, and 4 was 43, 15, 60, and 1, respectively.

**TABLE 1.** Characteristics of 119 hypercholesterolemic females

	Clinical Chemistry	Nightingale Metabolomics	Spearman Correlation Coefficients
LDL cholesterol (mmol/l)	5.25 ± 0.50	2.27 ± 0.26	0.66
Total cholesterol (mmol/l)	7.17 ± 0.64	5.57 ± 0.43	0.68
Triglyceride (mmol/l)	1.50 ± 0.68	1.45 ± 0.47	0.96
HDL cholesterol (mmol/l)	1.39 ± 0.28	1.47 ± 0.22	0.84
ApoB (g/l)	1.25 ± 0.14	1.10 ± 0.11	0.78

Data are expressed as mean ± SD; N = 119; Age (year), 32.90 ± 4.37; BMI, 27.9 ± 5.10.

**Fig. 2.**
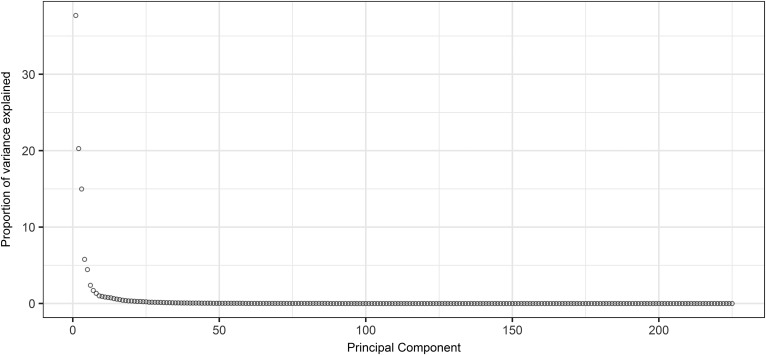
Proportion of variance explained by PCs derived from plasma metabolomics data of 119 hypercholesterolemic females.

**Fig. 3.**
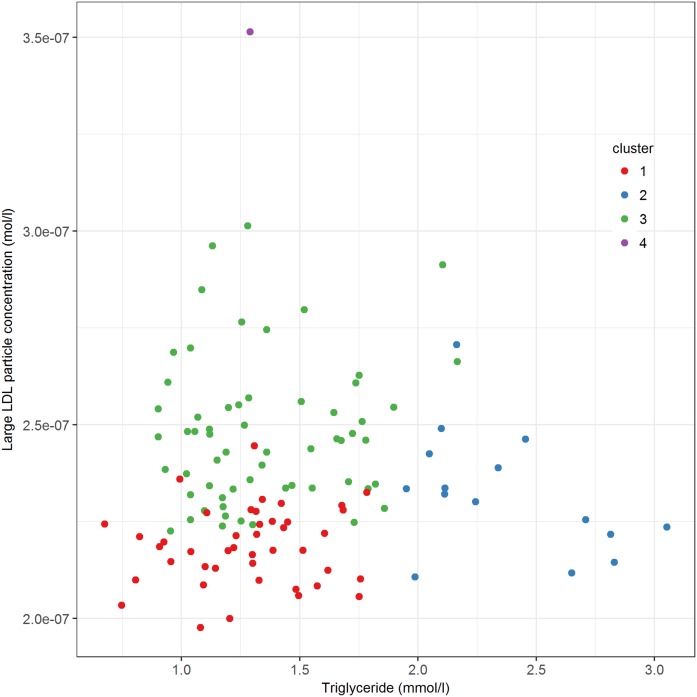
Plasma triglyceride against large LDL particle concentration in 119 hypercholesterolemic females. Different colors refer to the hierarchical clustering outcomes (red, cluster 1; blue, cluster 2; green, cluster 3; purple, cluster 4).

To analyze the divergence of the different clusters, we ran PCA. The first and second PC explained 38% and 21% of the total variance of the metabolic variables across the 119 individuals, respectively (**Fig. 2**). To understand which metabolites corresponded to the first and second PC the most, we calculated the Spearman correlation coefficients between original variables and PCs (supplemental Table S2). We observed that plasma triglyceride and large LDL particle concentration were the most correlated variables with the PC1 (Spearman correlation coefficient −0.988) and PC2 (Spearman correlation coefficient −0.978), respectively. Therefore, we used these two variables to represent the axes of PC1 and PC2 (**Fig. 3**). Our next question was whether the four clusters derived from the hierarchical clustering analysis (Fig. 1) were indeed separated by PC1 and PC2. To answer that, we added the hierarchical clustering results to the scatterplot (Fig. 3). Inspection reveals that the females in cluster 3 are separated from the other groups by showing a high plasma large LDL particle concentration coupled with relatively low plasma triglyceride, suggesting a defect in hepatic LDL uptake.

**Fig. 4.**
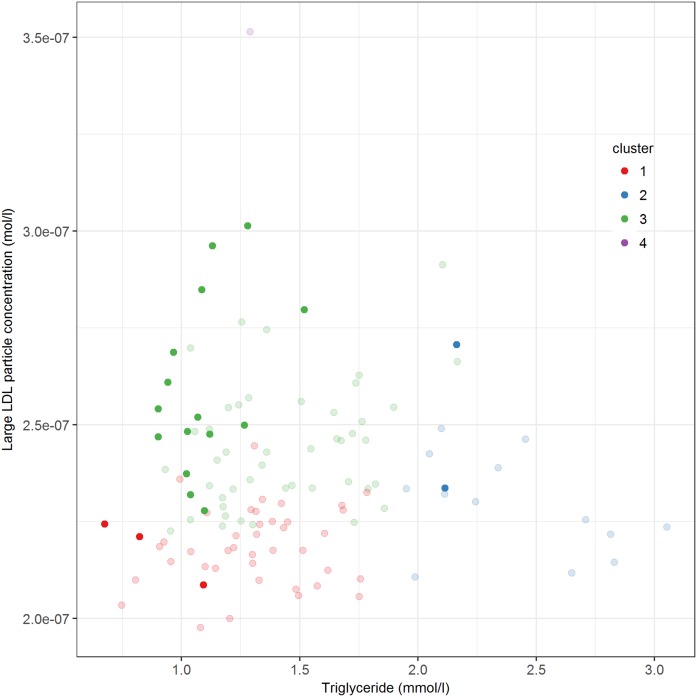
Plasma triglyceride against large LDL particle concentration in 119 hypercholesterolemic females. Different colors refer to the hierarchical clustering outcomes (red, cluster 1; blue, cluster 2; green, cluster 3; purple, cluster 4). The hypercholesterolemic females with mutations that were known to affect the LDLR pathway were highlighted.

**Fig. 5.**
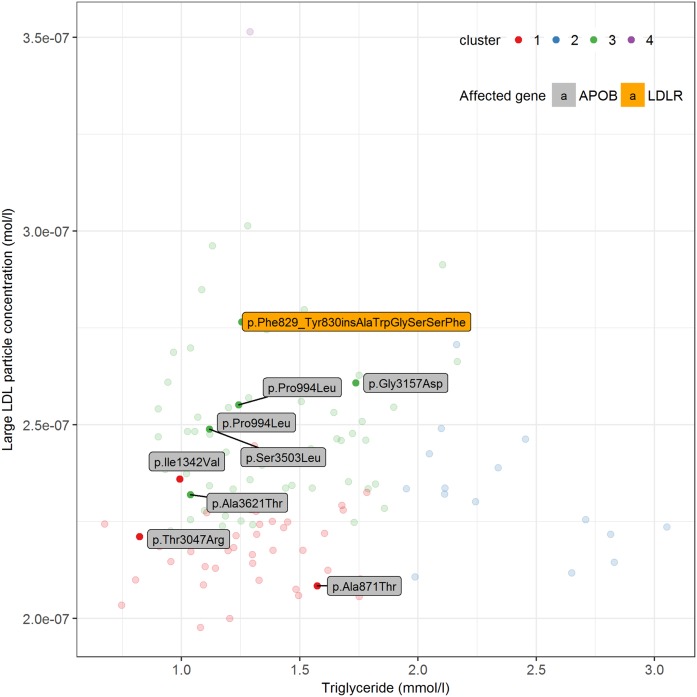
Plasma triglyceride against large LDL particle concentration in 119 hypercholesterolemic females. Different colors refer to the hierarchical clustering outcomes (red, cluster 1; blue, cluster 2; green, cluster 3; purple, cluster 4). The highlighted dots represent eight individuals who carry a heterozygous variant in *LDLR* or *APOB* of unknown clinical significance. The specific variant in *LDLR* or *APOB* is shown.

Because we sequenced *LDLR*, *APOB*, and *PCSK9* in all subjects, we could verify whether the females with known heterozygous mutations in the LDLR pathway plotted in the region of cluster 3. Indeed, from 20 subjects with heterozygous mutations in *LDLR* or *APOB*, 15 subjects were located in cluster 3 (**Fig. 4**). The other five carriers were found in cluster 1 (n = 3) and cluster 2 (n = 2). In addition, we identified eight women who were heterozygous carriers of a novel variant in *LDLR* or *APOB* from which the pathogenicity has not yet been determined. Five of these eight subjects were positioned in cluster 3 and three in cluster 1 (**Fig. 5**).

To improve our understanding of the underlying pathobiology of the elevated plasma LDL cholesterol in the remaining 91 women, we calculated the wGRS and lifestyle score, and assessed the associations between both scores and plasma concentrations of large LDL particles and triglyceride. As shown in supplemental Figs. S1 and S2, no relation could be demonstrated between both scores and plasma large LDL particle concentration (wGRS: Kendall tau correlation coefficient −0.017, *P* = 0.80; lifestyle score: Kendall tau correlation coefficient −0.04, *P* = 0.57). Both scores showed moderate association with plasma triglyceride concentration (wGRS: Kendall tau correlation coefficient −0.156, *P* = 0.02; lifestyle score: Kendall tau correlation coefficient −0.198, *P* = 0.0099).

## DISCUSSION

In the current study, we showed that combining plasma metabolomics data with genetic information can improve our understanding of the origin of severe hypercholesterolemia in young healthy women. These analyses may help with the diagnosis and personalized treatment of patients with hypercholesterolemia in which no causal mutations in the canonical LDL genes can be identified.

Metabolic profiling has been used in a large number of cohort studies to assess the value of circulating metabolites in prediction of risk for cardiovascular events (21, 22). More specifically, metabolomics has been used to study associations between circulating metabolites and statin usage (23), CETP inhibition (24), and PCSK9 inhibition (25), generating insight into the broad metabolic effects of these interventions. Nightingale metabolomics data contain not only concentrations in different units but also other quantities, such as ratios, percentages, degrees of saturation, and lipoprotein particle size. Therefore, in the current study, we scaled all the metabolic variables to make them have equal importance in the hierarchical clustering.

The hierarchical clustering analysis revealed four clusters in the 119 hypercholesterolemic females with plasma LDL cholesterol above the 99th percentile for their age. We hypothesized that mutations in genes belonging to the same metabolic pathway (e.g., the LDLR pathway) should render a similar plasma metabolome (one cluster). The PCA revealed that plasma triglyceride and large LDL particle concentrations were the major discriminators for the four clusters. Because cluster 3 is characterized by a high concentration of large LDL particles and relatively low triglyceride in plasma, we hypothesized that this cluster represented the hypercholesterolemia due to defective LDL clearance. Incorporation of the genetic information provided us with the verdict, because we expected the 20 subjects carrying a known functional heterozygous mutation in *LDLR* or *APOB* to position in cluster 3. Indeed, 15 subjects fit this hypothesis and were located in cluster 3.

Then we came up with the question: “Can we get insight into whether a novel variant in *LDLR* or *APOB* is the underlying cause for the severe hypercholesterolemia based on the metabolome profile?” Indeed, six out of eight carriers of a novel mutation fit in cluster 3, suggesting potential effects of these variants on LDLR-mediated uptake. This observation suggests that metabolic profiling is useful to delineate the subjects with a pathogenic mutation from those that do not carry any variant in either *LDLR* or *APOB*. However, not all subjects in cluster 3 carry a variant in *LDLR* or *APOB*. We realize that the pathway of LDLR-mediated endocytosis and intracellular cholesterol trafficking contains many more genes (26–28) than we have sequenced in our cohort. So expansion of the number of genes on the chip or choosing whole genome sequencing will ultimately improve the information on all genes involved in the LDLR pathway and may thus help to identify additional genetic variants underlying the pathobiology in the remaining 40 females in cluster 3. Meanwhile, we cannot exclude other processes underlying the hypercholesterolemia, such as epigenetic changes (12), lincRNA (29), microRNA (30), or combinations thereof.

Cluster 4 contained only one subject, and the individual had the highest large LDL particle concentration among the 119 hypercholesterolemic females. Interestingly, we did not identify any mutations in the sequenced genes, including *LDLR*, *APOB*, and *PCSK9*. This female subject was 28 years old with a BMI of 21.7 kg/m^2^. Her waist circumference was 69 centimeters. When we compared her plasma metabolomics data to the other 118 hypercholesterolemic females, we identified 77 outlier variables [either below the first quantile (1.5 × interquartile range) or above the third quantile (1.5 × interquartile range); supplemental Table S3]. We noticed that this female had a high proportion of esterified cholesterol in VLDL and HDL particles compared with the remaining 118 subjects. Interestingly, the CETPtg/apoCI^−/−^ mouse model showed a very similar phenotype (31). APOC1 is an important regulator for CETP activity, which may partly underlie the observed phenotype (32). So far, no mutations in *APOC1* have been described.

A recent study (33) showed that hypercholesterolemic subjects without any known genetic defect had lower levels of LDL cholesterol than those with a mutation. Therefore, we hypothesized that the origin of the hypercholesterolemia in cluster 1 may be either polygenic or due to lifestyle factors. After additional analysis of the relationships between the wGRS or lifestyle score and triglyceride or large LDL particle concentration, we observed that only genetic risk scores were negatively associated with triglyceride concentration (Kendall tau correlation coefficient −0.23, *P* = 0.04). This observation suggests that this cluster of hypercholesterolemic subjects may be caused by less damaging mutations in genes involved in the LDLR pathway. The major observation in the subjects located in cluster 2 is that they had elevated plasma triglyceride. The genetic array used in the current study does not contain the genes involved in triglyceride metabolism. Our data suggest that generation of a triglyceride-specific gene array may generate interesting results in the subjects in this cluster.

In summary, this study shows that bioinformatic analysis of metabolomics data derived from hypercholesterolemic subjects generates interesting clusters of patients that may help to guide targeted genomics approaches for hypercholesterolemia.

## Supplementary Material

Supplemental Data
