# Supplementary material for: Use of plasma metabolomics to analyze phenotype-genotype relationships in young hypercholesterolemic females
Source: J Lipid Res. 2018 Sep 28;59(11):2174–80. doi: 10.1194/jlr.M088930 (PMC6210900; doi:10.1194/jlr.M088930)
Supplement: Supplemental Data [file supp_59_11_2174__index.html]

Use of Plasma Metabolomics to Analyze Phenotype-Genotype Relationships in Young Hypercholesterolemic Females — Use of plasma metabolomics to analyze phenotype-genotype relationships in young hypercholesterolemic females — Supplemental Data 

# Use of plasma metabolomics to analyze phenotype-genotype relationships in young hypercholesterolemic females

## Supplemental Data

- Supplemental Table S1 (.xlsx, 24 KB) - Summary statistics of metabolic variables quantified by Nightinglae metabolomics platform.
- Supplemental Table S2 (.xlsx, 29 KB) - Spearman correlation coefficients between metabolic variables and principal components.
- Supplemental Table S3 (.xlsx, 16 KB) - Metabolic variables that were abnormal in the cluster 4 subject compared to the remaining 118 hypercholesterolemic females.
- Figure S1 (.jpg, 541 KB) - Scatterplots of weighted genetic risk score against triglyceride or large LDL particle concentration in 91 hypercholesterolemic females without canonical mutations.
- Figure S2 (.jpg, 482 KB) - Scatterplots of lifestyle score against triglyceride or large LDL particle concentration in 91 hypercholesterolemic females without canonical mutations.
