# Supplementary figures and images for: Use of plasma metabolomics to analyze phenotype-genotype relationships in young hypercholesterolemic females
Source: J Lipid Res. 2018 Sep 28;59(11):2174–80. doi: 10.1194/jlr.M088930 (PMC6210900; doi:10.1194/jlr.M088930)

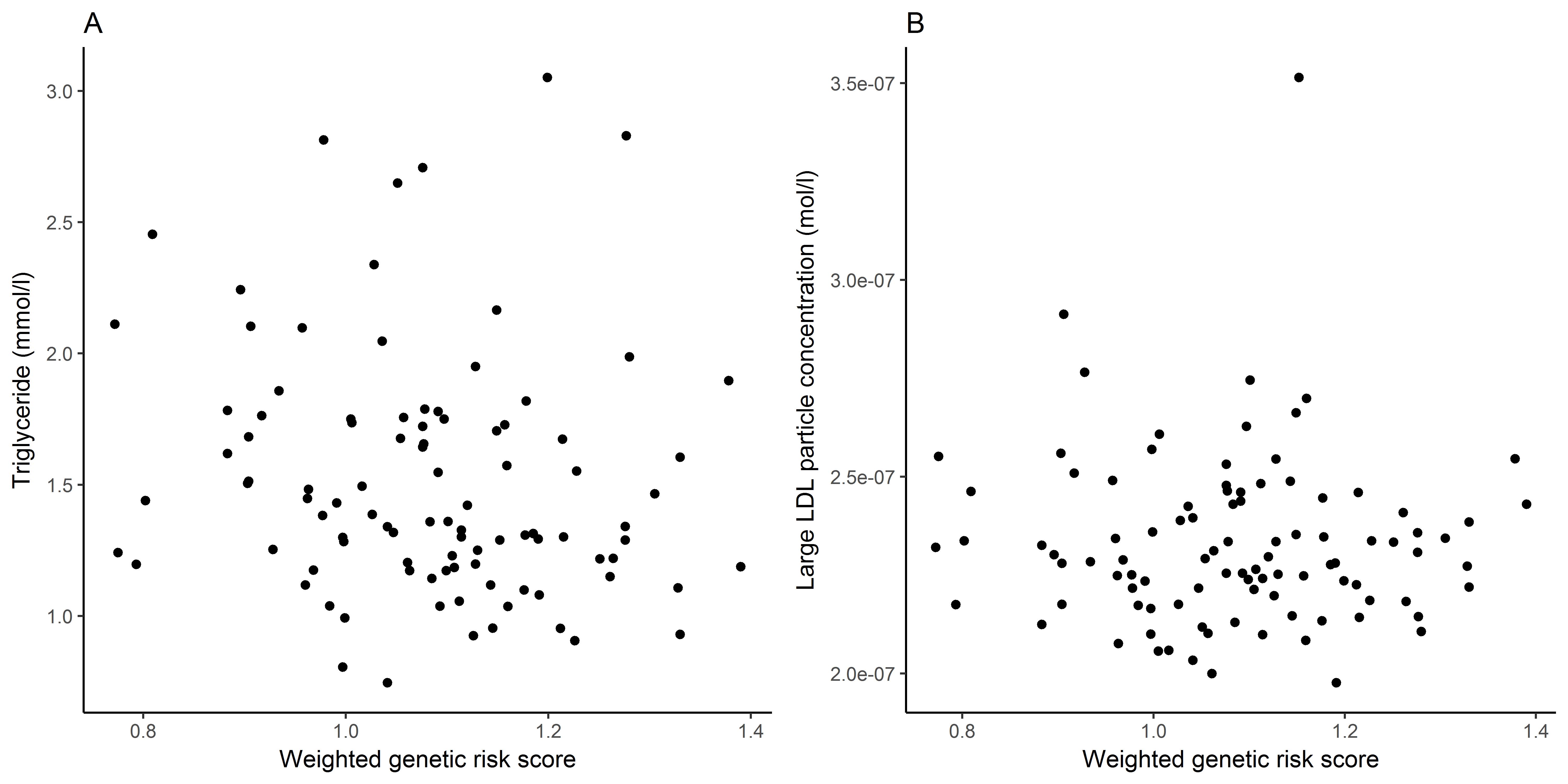

Supplement: Supplemental Data [file 10.1194_M088930_jlr.M088930-4.jpg]

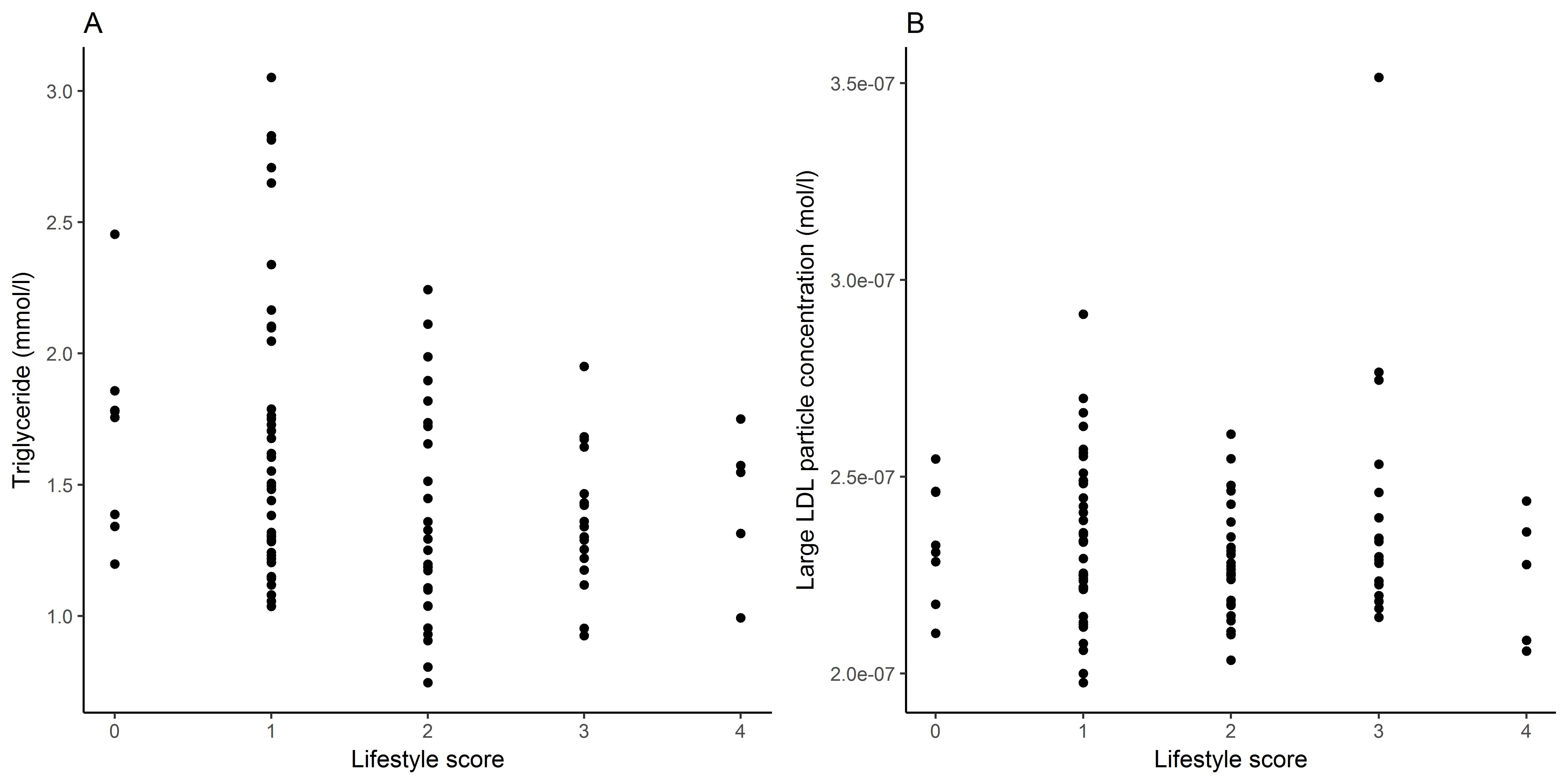

Supplement: Supplemental Data [file 10.1194_M088930_jlr.M088930-5.jpg]
